# Supplementary material for: Content-rich biological network constructed by mining PubMed abstracts
Source: BMC Bioinformatics. 2004 Oct 8;5:147. doi: 10.1186/1471-2105-5-147 (PMC528731; doi:10.1186/1471-2105-5-147)
Supplement: Additional File 2 — The original results of the above study (non-essential files are deleted to keep the file size under the limit set by BMC bioinformatics). [file 1471-2105-5-147-S2.bz2 › chilibotAdditionalFile2/dip05/676ID11323414E11082/html/TGFBR2_TGFBR3.html]

 


 **TGFBR2** and **TGFBR3** 
  
Found 4 abstracts in PubMed, retrieved 4.  
 

 What does Google say? 
 PDF only 
| .edu only 

---

**Interactive relationship** (e.g. stimulation, inhibition, etc)

**Neutral relationship**- Betaglycan  [ **TGFBR3** ]  was immunolocalized mainly to the epithelial cells in developing airways, a spatial distribution which overlaps with that of TGF beta type II receptor  [ **TGFBR2** ] .  Ref: 9739109 Mech Dev, 1998

**Non-interactive relationship** (e.g. studied together, co-existance, homology, etc.)

- These results suggest that the ALK5 SMAD4 part of the TGFB signalling pathway is a major target for inactivation in pancreatic carcinomas, that the expression of  **TGFBR2** ,  **TGFBR3** , and receptors involved in TGFB activation are maintained,.  Ref: 11408925 Int J Oncol, 2001
- We therefore studied the expression of UPA, UPAR, IGF2R, ALK5,  **TGFBR2** ,  **TGFBR3** , ENG, ALK1, TGFB1, TGFB2, and TGFB3 in a series of 14 pancreatic carcinoma cell lines.  Ref: 11408925 Int J Oncol, 2001
- These cells can also express TGF beta type II receptor  [ **TGFBR2** ]  and betaglycan  [ **TGFBR3** ] .  Ref: 9543637 Curr Eye Res, 1998
